# Evidence for differential alternative splicing in blood of young boys with autism spectrum disorders

**DOI:** 10.1186/2040-2392-4-30

**Published:** 2013-09-04

**Authors:** Boryana S Stamova, Yingfang Tian, Christine W Nordahl, Mark D Shen, Sally Rogers, David G Amaral, Frank R Sharp

**Affiliations:** 1 MIND Institute, University of California at Davis, Sacramento, CA 95817, USA; 2 Department of Neurology, University of California at Davis, Sacramento, CA 95817, USA; 3 Department of Psychiatry and Behavioral Sciences, University of California at Davis, Sacramento, CA 95817, USA; 4 College of Life Sciences, Shaanxi Normal University, Xi’an 710062, China; 5 MIND Institute Research Wet Labs, University of California at Davis, Room 2417, 2805 50th Street, Sacramento, CA 95817, USA

**Keywords:** Autism, ASD, RNA, Splicing, Head size, Gene expression

## Abstract

**Background:**

Since RNA expression differences have been reported in autism spectrum disorder (ASD) for blood and brain, and differential alternative splicing (DAS) has been reported in ASD brains, we determined if there was DAS in blood mRNA of ASD subjects compared to typically developing (TD) controls, as well as in ASD subgroups related to cerebral volume.

**Methods:**

RNA from blood was processed on whole genome exon arrays for 2-4–year-old ASD and TD boys. An ANCOVA with age and batch as covariates was used to predict DAS for ALL ASD (*n*=30), ASD with normal total cerebral volumes (NTCV), and ASD with large total cerebral volumes (LTCV) compared to TD controls (*n*=20).

**Results:**

A total of 53 genes were predicted to have DAS for ALL ASD *versus* TD, 169 genes for ASD_NTCV *versus* TD, 1 gene for ASD_LTCV *versus* TD, and 27 genes for ASD_LTCV *versus* ASD_NTCV. These differences were significant at *P* <0.05 after false discovery rate corrections for multiple comparisons (FDR <5% false positives). A number of the genes predicted to have DAS in ASD are known to regulate DAS (SFPQ, SRPK1, SRSF11, SRSF2IP, FUS, LSM14A). In addition, a number of genes with predicted DAS are involved in pathways implicated in previous ASD studies, such as ROS monocyte/macrophage, Natural Killer Cell, mTOR, and NGF signaling. The only pathways significant after multiple comparison corrections (FDR <0.05) were the Nrf2-mediated reactive oxygen species (ROS) oxidative response (superoxide dismutase 2, catalase, peroxiredoxin 1, PIK3C3, DNAJC17, microsomal glutathione S-transferase 3) and superoxide radical degradation (SOD2, CAT).

**Conclusions:**

These data support differences in alternative splicing of mRNA in blood of ASD subjects compared to TD controls that differ related to head size. The findings are preliminary, need to be replicated in independent cohorts, and predicted alternative splicing differences need to be confirmed using direct analytical methods.

## Background

Autism spectrum disorder (ASD) is a spectrum of neurodevelopmental disorders that are clinically defined by communication and social impairments combined with stereotypic and repetitive behaviors [1]. Though over 20% of those diagnosed with ASD have a number of identifiable genetic causes [2], the genetic bases for the remaining cases remain unclear. It is not known how many other genetic causes of ASD may be identified, or if there is a group of susceptibility genes that interact with environmental factors (for example, toxins, infection, immune) and cause the majority of cases of ASD [2-4].

A role for both genetics and environment in ASD has come from many studies, including a recent study of ASD brain. Regional patterns of gene expression that distinguish frontal and temporal cortex were attenuated in ASD brain compared to controls [5]. Moreover, there were two modules of co-expressed genes associated with autism including a neuronal module enriched for known autism susceptibility genes (genetic module); and a module enriched for immune and glial genes that were not enriched for autism GWAS-associated genes (environmental immune module) [5]. Significantly for the present study, some genes showed evidence of altered differential alternative splicing (DAS) of specific FOX1 regulated mRNAs [5].

These data led us to consider in this study whether there might be altered DAS of mRNA in ASD blood. Blood is useful to study in ASD for several reasons: (1) various factors - including genetics, toxicants, infections, immune and autoimmune factors implicated in ASD [6-11] - affect alternative splicing in leukocytes in blood [12-19]; (2) the monocyte transcriptome in blood is very similar to the microglia (brain macrophage) transcriptome [20,21], and microglia are activated in ASD brain [22]; (3) immune and autoimmune dysfunction that would be associated with alterations of gene expression and alternative splicing in blood has been reported in subgroups of ASD individuals [23-26]; (4) there is cross-talk between the immune system and the CNS [27,28]; (5) studies of blood allow us to investigate subjects at an early age when ASD becomes clinically evident.

In recruiting subjects for this study, we included ASD boys that were ultimately found to have altered trajectories of brain development. Abnormal brain enlargement has been consistently observed in MRI studies of preschool-aged ASD children compared to age-matched controls [29-31]. This is one of the most consistent findings in the neuropathology of a subset of ASD in boys, however there is considerable variability in total cerebral volume (TCV) phenotypes. Macrocephaly has been reported in 15% to 20% of young children with ASD [29,31-34] , though there is an ongoing debate for its relevance in ASD [35,36]. In the larger population of children recruited for this study, approximately 15% had abnormally enlarged TCV [30]. In addition, PTEN (phosphatase and tensin homolog deleted on chromosome 10) mutations are found in a subset of ASD children with macrocephaly [37-39]. It has been suggested that PTEN may regulate cell size through effects on protein translation, particularly interesting in the view of its connection to PI3K/AKT/mTOR signaling pathways implicated in single gene causes of ASD [40]. Finally, differences of differential alternative splicing have been associated with abnormal brain volumes in various animal models and in humans [41-44].

We therefore postulated that specific differences in alternative splicing/exon usage in immune blood cells may be present in ASD boys, and this might differ in ASD boys with large total cerebral volumes (ASD_LTCV) *versus* ASD boys with normal total cerebral volumes (ASD_NTCV). Thus, we compared ASD and ASD subgroups related to total cerebral volume to typically developing (TD) controls. Our findings demonstrate DAS in ASD *versus* TD boys, and that there are differences of DAS in ASD boys with large brains compared to those with normal total cerebral volumes. Finally, there are some genes that demonstrate DAS in blood in this study that have previously been reported to have DAS in brain [5]. However, most of the DAS blood genes are different from DAS brain genes which would be consistent with differential alternative splicing generally being tissue specific [45-48].

## Methods

### Subjects

This study was approved by the University of California at Davis Institutional Review Board (IRB). Written informed consent was obtained from the parent or guardian of each participant and data were analyzed without personal information identifiers.

Fifty boys (*n*=20 TD and *n*=30 ASD), aged 2-4 years, participated in this study. Participants were enrolled in the UC Davis MIND Institute’s Autism Phenome Project (APP) (http://www.ucdmc.ucdavis.edu/mindinstitute/research/app/). The diagnostic criteria for ASD and TD and inclusion and exclusion criteria were previously described [30]. Briefly, subjects were recruited through the MIND Institute at the University of California, Davis. ASD diagnosis was determined based on administration of the Autism Diagnostic Observation Schedule-Generic (ADOS-G) [49], and the Autism Diagnostic Interview-Revised (ADI-R) [50] by appropriately trained members of the research team and clinical consensus of two independent psychologists. Autism severity was determined by using the ADOS severity score, based on the algorithm scores on the various modules [51]. Developmental abilities of cases and controls were determined using the Mullen Scales of Early Development [52]. In addition, several inclusion and exclusion criteria were used for the TD group. TD inclusion was based on having Standard Score of 70-130 on the Learning Composite of the Mullens Scale of Early Learning, and a score <11 on the Social Communication Questionnaire used to screen for autism traits [53]. Exclusion criteria for the TD group included a diagnosis of intellectual disability, pervasive developmental disorder, specific language impairment, or any known developmental, neurological, or behavioral problems [30].

In addition, if the ASD or TD children had a fever or had been ill within the 2 weeks before the blood draw, blood draws were rescheduled for a later time. This method was used to exclude children with acute infections in a previous CHARGE study [54]. Clinical information collected for the kids from this study showed six were on different prescription and non-prescription medications (6 ASD/0 TD), and 25 (17 ASD/8 TD) were on supplements (mostly vitamins). Since no two ASD or TD individuals were on the same medication or supplement, and times varied since the last doses, these were not included as covariates in the analyses and there were too few medication- and supplement-free subjects to analyze separately.

### MRI of brain

Because total cerebral volume (TCV) is extremely variable in ASD [30], ASD participants were selected to have a range of TCV. As part of the APP, all participants underwent MRI scanning at the UC Davis Imaging Research Center, as previously described [30]. In short, all scans were acquired during natural, nocturnal sleep. Total cerebral volume (TCV) data were measured from a three-dimensional T1-weighted MPRAGE scan (TR, 2,170 ms; TE, 4.86 ms; matrix, 256 × 256; slices acquired in sagittal direction, 192; isotropic voxels, 1 mm). Distortion correction using a calibration phantom was carried out and images were corrected for field in homogeneity. TCV was measured using a template-based automated method, and excluded brainstem and cerebellum [30]. ASD subjects were divided into two subgroups based on MRI measures of TCV, with the ones with NTCV (*n*=20 ASD subjects) having TCV within 1.5 standard deviations of the TD centroid [30], while the ones with LTCV (*n*=10) had TCV >1.5 standard deviations of the TD controls.

### RNA and array processing

Whole venous blood was collected into PAXgene tubes (PreAnalytiX, Germany), which immediately lyse all cells in whole blood and stabilize the RNA without measurable degradation or *ex vivo* transcriptional changes. RNA was isolated and processed as previously described [55] using the WT-Ovation™ Pico RNA Amplification System (NuGEN, San Carlos, CA, USA) with the Exon Module and fragmented and labeled using the FL Ovation™ cDNA Biotin Module V2 (NuGEN, San Carlos, CA, USA). Expression was measured by hybridization on Affymetrix Human Exon 1.0 ST microarrays according to protocol (Affymetrix, Santa Clara, CA, USA).

Probe summarization and probe set normalization were performed using robust multi-chip average (RMA) in Partek Genomics Suite 6.5 software (Partek Inc., St. Louis, MO, USA). RMA includes background correction, Quantile Normalization, log_2_-transformation, and Median Polish probe set summarization. Only the core meta-probe sets (about 228,000 probe sets) were analyzed since these are the best annotated. Exons with low signal in all samples (maximum signal across all samples of the log_2_-transformed probe set value <3), were considered not expressed and were excluded from further analysis.

### Analysis of predicted DAS

Since the exon array used does not contain probe sets covering exon-exon junctions, deducing DAS is indirect given that the unit measured is exon expression which provides a measure of exon usage. Thus we will often use the term predicted ‘differential alternative splicing/differential exon usage (DAS/DEU)’ to emphasize the fact that differential exon expression is used to predict DAS in these studies.

An alternative splicing ANCOVA was performed on the probe sets passing the filtering criteria using Partek software. The Splicing ANCOVA Model [56,57] used was:

$$\begin{array}{c} Y_{\mathrm{ijklm}}=\mu +{\mathrm{Group}}_{i}+\mathrm{Age}+{\mathrm{Batch}}_{j}+{\mathrm{MarkerID}}_{k}\\ \;+\mathrm{SampleID}{\left(\mathrm{Group}*\mathrm{Batch}\right)}_{\mathrm{ijl}}\\ \;+\mathrm{Group}*{\mathrm{MarkerID}}_{\mathrm{ik}}+\mathrm{Age}*{\mathrm{MarkerID}}_{k}\\ \;+\mathrm{Batch}*{\mathrm{MarkerID}}_{\mathrm{jk}}+\epsilon_{\mathrm{ijklm}} \end{array}$$

Where:

•Y_ijklm_ represents the m^th^ observation on the i^th^ Group, j^th^ Batch, k^th^ MarkerID, l^th^ SampleID.

•μ is the common effect for the whole experiment.

•ϵ_ijklm_ represents the random error present in the m^th^ observation on the i^th^ Group, j^th^ Batch, k^th^ MarkerID, l^th^ SampleID. The errors ϵ_ijklm_ are assumed to be normally and independently distributed with mean 0 and standard deviation δ for all measurements.

SampleID(Group * Batch)_ijl_ is a sample-to-sample effect. SampleID is a random effect. Batch is a random effect.

MarkerID_k_ is exon-to-exon effect (alt-splicing independent to Group). This term also accounts for the fact that not all exons of a gene hybridize to the corresponding probe sets (MarkerID) with the same efficiency.

Group * MarkerID_ik_, Age * MarkerID_k_, Batch * MarkerID_jk_ represent whether an exon expresses differently in different level of the specified alternative splice factor(s). The term Group * MarkerID_ik_ was used to identify group-specific DAS.

Thus, an alternative splicing ANCOVA was performed on the 17,100 genes passing the filtering criteria (maximum signal across all samples of the log2-transformed probe set value <3, as described in the above section). The splicing ANCOVA model included covariates for both technical (batch, random effect) and biological (age, continuous variable) variation to account for their effect on DAS/DEU. In addition, since not all exons in a gene express/hybridize to the probe sets at the same level, MarkerID was added to the model to account for exon-to-exon differences. MarkerID is an exon-to-exon effect (alt-splicing independent of Group). SampleID was added to the model, which accounted for the sample-to-sample effect. An interaction term of MarkerID with Group (ASD, TD) to detect alternative splicing was used to estimate an exon has a different expression in different levels of the factor (ASD, TD), which was used to identify genes with predicted DAS/DEU in the different groups. The splicing prediction algorithm within Partek software was used.

Separate differential alternative splicing ANCOVAs were performed for the following comparisons: All ASD *versus* TD; ASD_LTCV *versus* TD; ASD_NTCV *versus* TD; and ASD_NTCV *versus* ASD_ LTCV. To correct for the multiple comparisons being performed, a Benjamini-Hochberg false discovery rate of 5% was adopted, meaning that the *P* values were adjusted so that no more than 5% of the reported genes would be expected to be false positives [58].

### Hierarchical clustering and principal component analyses

Hierarchical clustering using Euclidean distance and average linkage, and the principal components analysis (PCA) were performed in Partek Genomics Suite. For each analysis, the exon-level expression data corrected for age and batch were used. Genome-wide PCA using all probe sets on the array and all samples was performed to examine array quality and visualize variability at the whole-genome level (Additional file 1: Figure S1).

### Functional pathways associated with DAS in ASD

Ingenuity pathway analysis (IPA) was used to identify the pathways from the IPA library of canonical pathways that were most significant to each dataset. The significance of the association between the dataset with predicted DAS/DEU and canonical pathways was assessed by calculating the ratio of the number of genes from the dataset that map to the pathway divided by the total number of molecules that exist in the canonical pathway. A Fisher’s exact test was then used to calculate a *P* value determining the probability that each biological function and/or pathway assigned to that dataset is due to chance alone. A FDR (5% false positives) corrected *P* <0.05 was considered to be statistically significant for over-representation of the molecules in a given pathway. Thus, over-represented canonical pathways are those with more molecules than expected by chance.

The above analyses used standard criteria for identifying ASD pathways associated with DAS/DEU. However, as outlined in the results below, only two pathways were identified using the DAS/DEU genes that passed FDR correction. We therefore performed a sub-analysis in order to obtain a broader picture of possible regulated pathways. Thus, a splicing ANCOVA was performed for All ASD *versus* TD, except that all genes showing a *P* <0.05 for DAS were identified. An exon-level expression ANCOVA was then performed on these genes for ALL ASD *versus* TD, including age and batch as covariates. Exons with expression significantly different between All ASD and TD with *P* <0.005 and |Fold-Change| >1.2 were considered significant in this analysis. This approach would help correct for multiple comparisons and should identify the most reliable DAS genes because they were predicted to be differentially alternatively spliced, and to have significant differences of exon-level expression for All ASD *versus* TD (overlap *P* <0.0002). An Ingenuity pathway analysis was then performed on this list of genes for ALL ASD *versus* TD with *P* <0.05 considered significant.

## Results

### Participants’ characteristics

Demographic and clinical characteristics of the subjects are presented in Table 1. There were 20 ASD_NTCV subjects (3.0 ± 0.5 years), 10 ASD_LTCV subjects (3.1 ± 0.2 years), and 20 TD subjects (3.0 ± 0.3 years). Seven out of the 10 ASD_LTCV subjects were classified as megalencephalic, defined as having TCV >2 standard deviations above the TD mean. There were no significant group differences in age or race. As expected, developmental quotient (DQ), verbal quotient (VQ), and nonverbal quotient (NVQ) were significantly lower in the ASD than the TD groups (*P* <1E-09). Total cerebral volume (TCV) was not significantly different between the ASD_NTCV and TD groups (*P*=0.87). TCV was significantly enlarged in the ASD_LTCV compared to ASD_NTCV (*P*=2.42E-10) and TD (*P*=1.42E-09), which was the result of our pre-selection. There were no significant differences in the ADOS severity scores between the ASD_NTCV and ASD_LTCV groups (*P*=0.17).

**Table 1:** Demographic and clinical characteristics of autism spectrum disorder (ASD) and typically developing (TD) participants

	**TD**	**ASD**	**ASD Normal TCV**	**ASD Enlarged TCV**
*n*				
Subjects	20	30	20	10
Age (years)				
Mean ± SD	3.0 ± 0.3	2.9 ± 0.4	3.0 ± 0.5	3.1 ± 0.2
Gender				
Male, *n* (%)	20 (100%)	30 (100%)	20 (100%)	10 (100%)
Race, *n* (%)				
White	14 (70%)	15 (50%)	11(55%)	4 (40%)
Black	0 (0%)	3 (10%)	2 (10%)	1 (10%)
Asian	0 (0%)	1 (3%)	1 (5%)	0 (0%)
Other	0 (0%)	1 (3%)	1 (5%)	0 (0%)
Mixed	3 (15%)	2 (7%)	1(5%)	1 (10%)
Unknown	3 (15%)	8 (27%)	4 (20%)	4 (40%)
TCV (cm3)				
Mean ± SD	994.8 ± 65.0	1049.7 ± 87.4	997.7 ± 51.0	1153.7 ± 33.6
ADOS Severity Score				
Mean ± SD	NA	8.0 ± 1.7	8.3 ± 1.7	7.4 ± 1.6
Developmental quotient (DQ)				
Mean ± SD	106.6 ± 14.0	60.2 ± 22.7	61.4 ± 20.9	57.7 ± 27.0
Verbal quotient (VQ)				
Mean ± SD	109.5 ± 15.6	53.6 ± 28.2	54.4 ± 25.9	52.1 ± 33.8
Nonverbal quotient (NVQ)				
Mean ± SD	103.6 ± 14.6	66.7 ± 19.9	68.4 ± 18.9	63.3 ± 22.5

### DAS/DEU in ASD groups compared to TD

**Figure 1:**
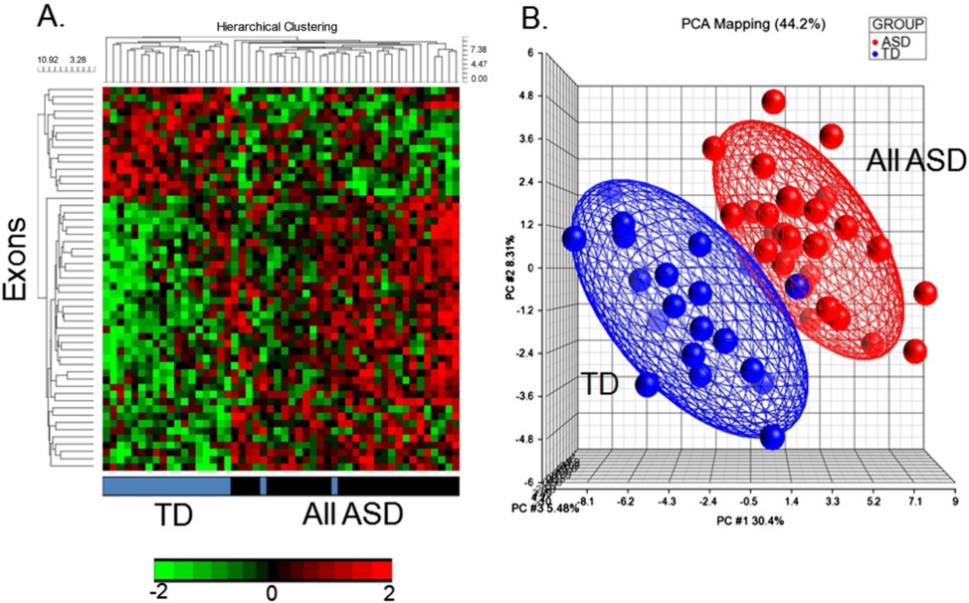
**All ASD *vs. *TD.** Unsupervised hierarchical clustering **(A)** and PCA **(B)** of the 53 exons predicted to display DAS/DEU with FDR *P* <0.05 in All ASD *vs.* TD analysis. The heatmap in 1A represents adjusted exon expression level (high=red, low=green) of the exons predicted to have DAS/DEU. TD subjects denoted in blue; ASD subjects - in black. The PCA plot in 1B shows separation of the ASD (red spheres) from the TD (blue spheres) subjects. The ellipsoids are drawn at 2 standard deviations around the group centroids.

There were 53 genes with predicted DAS/DEU for the comparisons of ALL ASD *versus* TD (Table 2, Additional file 2: Table S1) (FDR corrected *P* <0.05). The cluster analysis (Figure 1A) and Principal Components Analysis (Figure 1B) using these genes demonstrated good separation of the subjects in each group with a few exceptions (Figure 1).

**Table 2:** **Genes ( *n *=53) with predicted DAS/DEU in All ASD *vs. *TD groups (FDR *P *<0.05)**

**Gene symbol**	**Gene title**	**FDR *P *(DAS)**
CDK13	Cyclin-dependent kinase 13	5.50E-05
USP48	Ubiquitin specific peptidase 48	1.11E-03
SFPQ	Splicing factor proline/glutamine-rich	1.11E-03
FXR1	Fragile X mental retardation, autosomal homolog 1	1.11E-03
C19orf6	Chromosome 19 open reading frame 6	1.85E-03
CAT	Catalase	1.85E-03
ZNF330	Zinc finger protein 330	1.85E-03
C19orf2	Chromosome 19 open reading frame 2	1.85E-03
TARS2	Threonyl-tRNA synthetase 2, mitochondrial (putative)	3.66E-03
LRPPRC	Leucine-rich PPR-motif containing	3.86E-03
PIK3C3	Phosphoinositide-3-kinase, class 3	4.65E-03
CLTB	Clathrin, light chain B	5.13E-03
SOD2	Superoxide dismutase 2, mitochondrial	6.11E-03
OS9	Osteosarcoma amplified 9, endoplasmic reticulum lectin	6.11E-03
ACPT	Acid phosphatase, testicular	6.71E-03
PPP2R2A	Protein phosphatase 2, regulatory subunit B, alpha	1.05E-02
C14orf159	Chromosome 14 open reading frame 159	1.05E-02
FGR	Gardner-Rasheed feline sarcoma viral (v-fgr) oncogene homolog	1.09E-02
GSN	Gelsolin	1.09E-02
EAPP	E2F-associated phosphoprotein	1.22E-02
PIP4K2A	Phosphatidylinositol-5-phosphate 4-kinase, type II, alpha	1.24E-02
TADA3	Transcriptional adaptor 3	1.65E-02
PRSS36	Protease, serine, 36	2.17E-02
HELQ	Helicase, POLQ-like	2.17E-02
EMD	Emerin	2.47E-02
C1orf175	Chromosome 1 open reading frame 175	2.77E-02
AEBP2	AE binding protein 2	2.86E-02
R3HDM1	R3H domain containing 1	2.96E-02
SRPK1	Serine/arginine-rich splicing factor kinase 1	3.29E-02
LEF1	Lymphoid enhancer-binding factor 1	3.29E-02
MPHOSPH10	M-phase phosphoprotein 10 (U3 small nucleolar ribonucleoprote	3.29E-02
PRDX1	Peroxiredoxin 1	3.29E-02
MPP1	Membrane protein, palmitoylated 1, 55kDa	3.29E-02
CNOT2	CCR4-NOT transcription complex, subunit 2	3.53E-02
GOLGA7	Golgin A7	3.53E-02
WDR67	WD repeat domain 67	3.61E-02
AAMP	Angio-associated, migratory cell protein	4.10E-02
KLHL9	Kelch-like 9 (Drosophila)	4.24E-02
CC2D1A	Coiled-coil and C2 domain containing 1A	4.49E-02
STAT4	Signal transducer and activator of transcription 4	4.49E-02
DHX29	DEAH (Asp-Glu-Ala-His) box polypeptide 29	4.51E-02
MGST3	Microsomal glutathione S-transferase 3	4.51E-02
TEPP	Testis, prostate, and placenta expressed	4.51E-02
UTRN	Utrophin	4.51E-02
PUM2	Pumilio homolog 2 (Drosophila)	4.51E-02
CHID1	Chitinase domain containing 1	4.51E-02
GFER	Growth factor, augmenter of liver regeneration	4.51E-02
RPGR	Retinitis pigmentosa GTPase regulator	4.51E-02
SUCLA2	Succinate-CoA ligase, ADP-forming, beta subunit	4.72E-02
ZNF512B	Zinc finger protein 512B	4.72E-02
MORN2	MORN repeat containing 2	4.72E-02
DNAJC17	DnaJ (Hsp40) homolog, subfamily C, member 17	4.88E-02
FGD3	FYVE, RhoGEF and PH domain containing 3	4.88E-02

**Figure 2:**
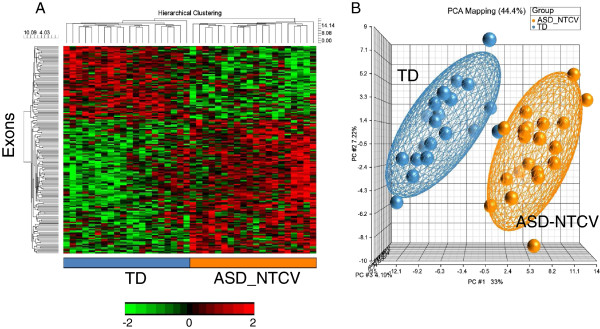
**ASD_NTCV *vs. *TD.** Unsupervised hierarchical clustering **(A)** and PCA **(B)** of the 169 exons predicted to display DAS/DEU with FDR *P* <0.05 in ASD_NTCV *vs.* TD analysis. The heatmap in 1A represents adjusted exon expression level (high=red, low=green) of the exons predicted to have DAS/DEU. TD subjects denoted in blue; ASD_NTCV subjects - in orange. The PCA plot in 1B shows separation of the ASD_NTCV (orange spheres) from the TD (blue spheres) subjects. The ellipsoids are drawn at 2 standard deviations around the group centroids.

There were 169 genes with predicted DAS/DEU for the comparisons of ASD_NTCV *versus* TD (FDR corrected *P* <0.05) (Additional file 2: Table S2). The cluster analysis (Figure 2A) and principal components analysis (Figure 2B) using these genes demonstrated excellent separation of the subjects in each group with no exceptions (Figure 2).

There was only one gene with predicted DAS/DEU for the comparison of ASD_LTCV *versus* TD (FDR corrected *P* <0.05). This was C19orf6 (Chromosome 19 open reading frame 6) which is also called membralin (MBRL).

**Figure 3:**
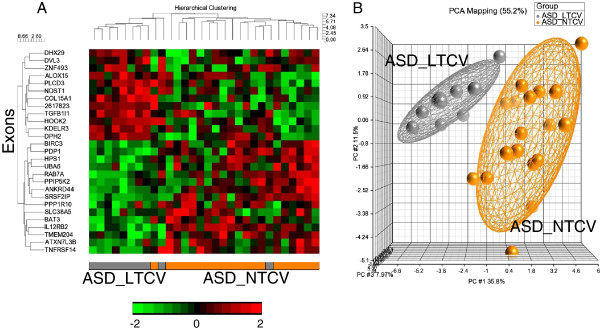
**ASD_LTCV *vs. *ASD_NTCV.** Unsupervised hierarchical clustering **(A)** and PCA **(B)** of the 27 exons predicted to display DAS/DEU with FDR *P* <0.05 in ASD_LTCV *vs.* ASD_NTCV analysis. The heatmap in 1A represents adjusted exon expression level (high=red, low=green) of the exons predicted to have DAS/DEU. ASD_LTCV subjects denoted in grey; ASD_NTCV subjects - in orange. The PCA plot in 1B shows separation of the ASD_NTCV (orange spheres) from the TD (grey spheres) subjects. The ellipsoids are drawn at 2 standard deviations around the group centroids.

There were 27 genes with predicted DAS/DEU for the comparisons of ASD_LTCV *versus* ASD_NTCV (FDR corrected *P* <0.05) (Table 3, Additional file 2: Table S3). The cluster analysis (Figure 3A) and principal components analysis (Figure 3B) using these 27 genes demonstrated excellent separation of the subjects in each group (Figure 3).

**Table 3:** **Genes ( *n *=27) with predicted DAS/DEU in ASD_LTCV *vs. *ASD_NTCV groups (FDR *P *<0.05)**

**Gene symbol**	**Gene title**	**FDR *P *(DAS)**
TMEM204	Transmembrane protein 204	2.26E-05
SRSF2IP	Splicing factor, arginine/serine-rich 2-interacting protein	7.17E-05
PPP1R10	Protein phosphatase 1, regulatory (inhibitor) subunit 10	4.19E-04
ATXN7L3B	Ataxin 7-like 3B	1.52E-03
HPS1	Hermansky-Pudlak syndrome 1	1.90E-03
ANKRD44	Ankyrin repeat domain 44	4.46E-03
EXOG	Endo/exonuclease (5'-3'), endonuclease G-like	5.11E-03
ZNF493	Zinc finger protein 493	1.51E-02
DHX29	DEAH (Asp-Glu-Ala-His) box polypeptide 29	1.57E-02
HOOK2	Hook homolog 2 (Drosophila)	2.02E-02
IL12RB2	Interleukin 12 receptor, beta 2	2.14E-02
BAT3	HLA-B associated transcript 3	2.53E-02
DVL3	Dishevelled, dsh homolog 3 (Drosophila)	2.53E-02
RAB7A	RAB7A, member RAS oncogene family	2.53E-02
TNFRSF14	Tumor necrosis factor receptor superfamily, member 14	2.53E-02
PPIP5K2	Diphosphoinositol pentakisphosphate kinase 2	2.67E-02
NDST1	N-deacetylase/N-sulfotransferase (heparan glucosaminyl) 1	3.26E-02
DPH2	DPH2 homolog (S. cerevisiae)	3.71E-02
PDP1	Pyruvate dehyrogenase phosphatase catalytic subunit 1	3.71E-02
TGFB1I1	Transforming growth factor beta 1 induced transcript 1	4.14E-02
UBA6	Ubiquitin-like modifier activating enzyme 6	4.41E-02
SLC38A5	Solute carrier family 38, member 5	4.42E-02
PLCD3	Phospholipase C, delta 3	4.45E-02
COL15A1	Collagen, type XV, alpha 1	4.47E-02
KDELR3	KDEL (Lys-Asp-Glu-Leu) endoplasmic reticulum protein	4.47E-02
BIRC3	Baculoviral IAP repeat-containing 3	4.47E-02
ALOX15	Arachidonate 15-lipoxygenase	4.47E-02

### Pathway analyses

Pathway analysis on each of the above lists of genes showed that only two pathways were significantly different at *P* <0.05 after FDR correction for multiple comparisons (FDR <5% false positives). These two pathways were for the 53 genes for ALL ASD *versus* TD comparison and included: (1) the Nrf2-mediated oxidative stress response (SOD2, CAT, PRDX1, PIK3C3, DNAJC17, and MGST3); and (2) the superoxide radical degradation (SOD2, CAT). This pathway analysis was supplemented by a network analysis which considered all of the up- and downregulated FDR passing genes and included direct as well as indirect interactions. The number one network for All ASD *versus* TD was: Free Radical Scavenging, Cell Death and Survival, Small Molecule Biochemistry (Additional file 1: Figure S2), which had PI3K as one of the central hubs. Notably, the second highest scoring network in ASD_LTCV *versus* ASD_NTCV included genes involved in RNA post-transcriptional modification with Ubiqiutin C as a central hub of direct interactions (Additional file 1: Figure S3).

As described in the Methods section, we performed a sub-analysis to identify additional pathways associated with ASD *versus* TD. There were 477 genes predicted to have DAS between ALL ASD *versus* TD (*P* <0.05) and that had significant differences in exon expression between ASD and TD (*P* <0.005) and a fold change of at least 1.2 or more (Additional file 2: Table S4). IPA analysis of these 477 genes revealed 21 pathways that were significantly different for ALL ASD *versus* TD (*P* <0.05) (Additional file 2: Table S5).

**Figure 4:**
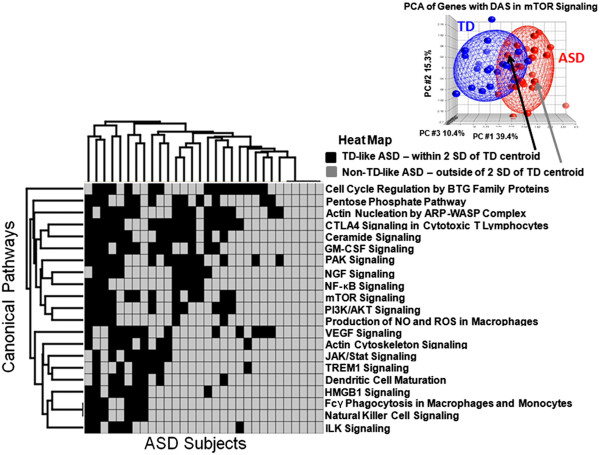
**Individual-subject level analysis.** Hierarchical clustering of ASD subjects and significant pathways (*P* <0.05). Each ASD subject in each of the significant pathways was scored as either TD-like, if its PCA position on the PCA plot for the genes with DAS/DEU in the particular pathway was within 2 SD of the TD centroid (within the TD ellipsoid); or as non-TD-like, if its PCA position on the first three PCA components space was outside 2 SD of the TD centroid (outside of the TD ellipsoid). An example of the scoring scheme - in the upper right-hand corner - is presented for the mTOR signaling pathway.

We then determined which of the 21 pathways were dysregulated in each ASD individual. To do this unique analysis, PCA mapping was performed based on exon-level expression of the genes predicted to have DAS/DEU in each pathway found to be over-represented with genes with DAS/DEU. For example, the PCA plot of the mTOR signaling pathway (Figure 4, upper right inset) was based on the expression level of eight exons in mTOR genes predicted to have DAS/DEU (Additional file 2: Table S5). Based on the PCA position of each ASD subject, the mTOR signaling pathway was scored as TD-like (if within 2 standard deviations of the TD centroid), or non-TD-like (if outside the 2 standard deviations of the TD centroid) (Figure 4, upper right inset). The same procedure was then performed for each of the 21 significantly over-represented canonical pathways, with each individual being assigned either as being ‘TD-like ASD’ (black rectangles) or ‘non-TD-like ASD’ (gray rectangles) for each pathway (Figure 4). Hierarchical clustering of the ASD subjects and the 21 canonical pathways (Figure 4) showed that different combinations of pathways were affected (non-TD-like ASD, gray rectangles) in different ASD subjects (Figure 4). No pathway was affected in every subject. Five ASD subjects had all 21 pathways affected (Figure 4, right side of x-axis cluster), but there were some ASD subjects with only several pathways affected (Figure 4, left side of x-axis cluster). The pathways affected in the majority of the ASD subjects included ILK Signaling, Natural Killer Cell Signaling, FCγ Receptor-mediated phagocytosis in macrophages and monocytes, and HMGB1 signaling (Figure 4, lower portion of cluster). The mTOR pathway was altered in 18 out of 30 ASD subjects (Figure 4). We performed this clustering analysis not so much to show clustering of pathways, since a number of these pathways contained common genes with DAS, but to identify clustering of subjects with particular sets of pathways being affected.

## Discussion

This study shows that differential alternative splicing may occur in selected genes in blood of 2-4-year-old boys with ASD compared to TD controls. There was an over-representation of DAS genes for pathways associated with the Nrf2-mediated oxidative stress response and superoxide radical degradation. In addition, there were genes with DAS from the Natural Killer cell, monocyte, NGF, and mTOR signaling pathways, which have been implicated in previous ASD studies [38,40,59-62]. An interesting result was that DAS in blood of ASD boys appeared to be more associated with normal cerebral volumes than with large cerebral volumes. Though the data are limited by small sample size and dependence of DAS predictions from exon arrays, they provide support for DAS occurring in blood as well as that already reported in brain [5], and will provide candidate genes for subsequent confirmatory studies.

### Oxidative stress responses

DAS was predicted for specific genes in the superoxide radical degradation and Nrf-2 mediated oxidative stress pathways which were significant for the ALL ASD *versus* TD comparison. SOD2 is a mitochondrial matrix protein that transforms toxic superoxide, a by-product of the mitochondrial electron transport chain, into hydrogen peroxide and diatomic oxygen. Mice without SOD2 die shortly after birth [63]. Catalase decomposes toxic hydrogen peroxide produced by SOD to water and oxygen. Peroxiredoxin 1 (PRDX1) catalyzes peroxide reduction of hydrogen peroxide, organic hydroperoxides, and peroxynitrite, and thus decreases oxidative stress in cells. The MGST3 gene encodes the enzyme Microsomal glutathione S-transferase 3 which demonstrates glutathione-dependent peroxidase activity towards lipid hydroperoxides. All of these genes are induced by reactive oxygen species (ROS). ROS cause the transcription factor Nrf2 to translocate from cytoplasm to the nucleus where Nrf2 binds anti-oxidant response elements (ARE) in promoters of Nrf2 target anti-oxidant genes including SOD2, CAT, PRDX1, and MGST3 to increase RNA expression [64].

There is some evidence of oxidative stress [65-68] and abnormal levels of superoxide and catalase in blood of ASD children [69-71]. There is also increased oxidative stress in ASD brain and specific abnormalities related to glutathione and superoxide [67,72]. Moreover, mitochondrial abnormalities have been identified in blood of ASD subjects [9] that could contribute to ROS production and alterations of Nrf2 and SOD2 pathways identified here. Mitochondrial abnormalities are also found in brain [73]. Though oxidative stress may not be causal, the results of the current study support the possibility of DAS in specific oxidative stress genes in peripheral blood cells is associated with ASD. This association probably is not specific since evidence of oxidative stress is seen in other neurodevelopmental disorders and also occurs during normal function of the immune system.

### Regulators of alternative splicing

A number of the genes predicted to have DAS in ASD either regulate splicing and/or are transcriptional regulators. Some of these genes included: SFPQ (splicing factor proline/glutamine rich), SRPK1 (serine/arginine-rich splicing factor kinase 1), SRSF11 (serine/arginine-rich splicing factor 11), SRSF2IP (splicing factor, arginine/serine-rich 2-interacting protein), FUS (fused in sarcoma), and LSM14A (SCD6 homolog A-yeast).

SFPQ , a DNA and RNA binding protein, is an essential pre-mRNA splicing factor required early in spliceosome formation and for splicing catalytic step II. It binds to pre-mRNA in the spliceosome C complex and regulates both alternative splicing and transcription. SFPQ has been associated with Alzheimer’s disease [74] and plays a role in neuronal survival and differentiation during development [75].

SRPK1 regulates splicing, controlling the intranuclear distribution of splicing factors in interphase cells, and regulates splice site selection. Alternative splicing of this gene results in multiple transcript variants. In brain, SRPK1 is expressed in cortical and hippocampal pyramidal neurons, cortical and cerebellar granule cells, and Purkinje cell neurons, and regulates alternative splicing of glutamate receptor subunit 2 (GluR2) and tau protein [76].

The SRSF11 gene encodes a nuclear protein that contains an arginine/serine-rich region similar to segments found in other pre-mRNA splicing factors and also plays a role in pre-mRNA processing and splicing including the human telomerase protein [77]. The related SRSF2IP (SCAF11) is another pre-mRNA splicing factor showing differential alternative splicing in ASD in this study.

The Fus gene encodes a protein component of the heterogeneous nuclear ribonucleoprotein (hnRNP) complex which regulates pre-mRNA splicing and export of fully processed mRNA to cytoplasm. This protein belongs to the FET family of RNA-binding proteins which regulate gene expression and process mRNA/microRNA. Fus proteins are found in brain cytoplasmic inclusions of patients with fronto-temporal dementia, affect tau splicing, and Fus mutations have been associated with familial amyotrophic lateral sclerosis [78]. Fus protein decreases in cortex during development [79].

LSM14A is homologous to Sm-like (LSm) proteins which are members of the tri-snRNP (small nuclear ribonucleoprotein) particles that regulate pre-mRNA splicing. Mutations in snRNP proteins have been associated with neurodevelopmental disorders with ASD features, including Prader-Willi and Angelman Syndrome [80,81].

Though most of the above genes have not been directly implicated in ASD, they could affect differential alternative splicing of ASD susceptibility genes [82]. Moreover, alternative splicing appears to play a role in some known genetic causes of ASD. There is aberrant alternative splicing in single gene mutations associated with ASD (*CADPS2*, *NLGN3*, *NLGN4X*, *NRXN1*, and *SHANK3*). There is dysregulated splicing in some single gene disorders responsible for some forms of syndromic autism (tuberous sclerosis and neurofibromatosis); and still other genes associated with syndromic autisms encode proteins that can modulate alternative splicing (*FMR1*, *MECP2*, and *SNRPN-*Fragile X and Retts Syndrome) [82]. In addition, a recent study demonstrated differential expression of mRNA in brain between ASD and controls, which revealed enrichment of mRNAs involved in the process of alternative splicing [35].

### Regulation of transcription, protein synthesis, and protein degradation

The number two network for ASD_NTCV *versus* TD was Cellular Assembly, Function and Organization and RNA Post-Transcriptional Modification which had Ubiquitin C at its hub (Additional file 1: Figure S2). There were a number differentially alternatively spliced genes in our ASD analyses that functioned in regulating transcription, translation, and protein degradation at various levels.

### Transcription

There were many genes with predicted DAS in ASD *versus* TD that regulated transcription including POLR2B (polymerase (RNA) II (DNA directed) polypeptide B, 140kDa). POLR2B encodes the second largest subunit of RNA polymerase II, the polymerase responsible for synthesizing messenger RNA in eukaryotes. This subunit, in combination with at least two other polymerase subunits, forms a structure within the polymerase that maintains contact in the active site of the enzyme between the DNA template and the newly synthesized RNA, playing a key role in transcription as well as alternative splicing. Though not implicated in ASD, in Alzheimer brain the hyperphosphorylation of RNA polymerase II and decreases of RNA levels precede neurofibrillary tangle formation [83].

MED12 (mediator complex subunit 12) is a component of the Mediator complex, a co-activator involved in the regulated transcription of nearly all RNA polymerase II-dependent genes. This subunit may regulate transcription of targets of the Wnt signaling pathway and SHH signaling pathway relevant for neurodevelopment and intellectual disability syndromes [84-86].

Another DAS gene was TADA3 (Transcriptional Adaptor 3) which enhances the initiation rate of RNA polymerase II-mediated gene transcription by interacting functionally with the general transcription machinery bound at the basal promoter (see below). Related genes included MLL3, a histone methyl transferease, which is a co-activator complex of nuclear receptors, involved in transcriptional co-activation. The gene is essential for normal embryogenesis and cell cycle progression [87]. Another related DAS gene is ASH2L (ash2 (absent, small, or homeotic)-like (Drosophila) is part of the MLL1/MLL2 histone methyltransferase (HMT) complex. It is involved in methylation/ demethylation and functions as a transcriptional activator and may play a role in chromosome demarcation [88].

There were also several transcription factors predicted to have DAS in ASD including STAT4, CNOT2, and NPAS2 and TCF1 (Transcription Factor 19). NPAS2, a member of the basic helix-loop-helix (bHLH)-PAS family of transcription factors, regulates transcription of proteins involved in specific types of memory, several proteins (PER1) that are part of the molecular clock in the mammalian forebrain, and has been implicated in ASD [89]. STAT4 (signal transducer and activator of transcription 4) is essential for mediating responses to IL12 in lymphocytes, and regulating the differentiation of T helper cells. STAT4 regulates perforin expression in cytotoxic T cells and Natural Killer cells [90], the latter cells having been shown to have altered cytotoxic/perforin functions in ASD [59].

There is evidence for coupling of transcription and splicing, with faster rates of transcription being associated with exon skipping [91,92]. Thus DAS in genes affecting transcription could result in alterations of DAS in a number of targets.

### Translation

There were also many DAS genes involved in regulating translation including: PIK3C3 (in mTOR pathway); PPP1R9B ( protein phosphatase 1, regulatory (inhibitor) subunit 9B); FXR1 (fragile X mental retardation, autosomal homolog 1); PPP2R2A (protein phosphatase 2, regulatory subunit B, alpha-mTOR); ELP2 (elongation protein 2 homolog-yeast); EIF2AK1 (eurkaryotic translation initiation factor 2-alpha kinase 1); MRPL40 (mitochondrial ribosomal protein L 40); TARS2 (threonyl-tRNA synthetase 2, mitochondrial); YARS (tyrosyl -tRNA synthetase); and EIF2C3 (eukaryotic translation initiation factor 2C, 3). These many genes involved in translation relate directly and indirectly to PI3K/AKT/mTOR pathways, a result which was also supported by our sub-analysis on the pathways affected in each ASD subject discussed below (see section on Pathways previously implicated in ASD).

### Ubiquitination and protein degradation

Several genes with predicted DAS in ASD were related to ubiquitin pathways, and ubiquitin was a hub in several regulated networks (Additional file 1: Figure S2). These genes included USP48 (ubiquitin specific peptidase 48), UBA6 (ubiquitin-like modifier activating enzyme 6), BIRC3 (baculoviral IAP repeat-containing 3), and DnaJC17 (DNAJ (Hsp40) homolog, subfamily C, member 17). BIRC3 Acts as an E3 ubiquitin-protein ligase regulating NF-kappa-B signaling and regulates both canonical and non-canonical NF-kappa-B signaling. The target proteins for its E3 ubiquitin-protein ligase activity include: RIPK1, RIPK2, RIPK3, RIPK4, CASP3, CASP7, CASP8, TRAF1, and BCL10. Ubiqutin pathways have been implicated in autism for some time and are specifically linked to autism associated with 15q11-q13 chromosome deletions [93,94].

### Genes implicated in previous autism studies - GWAS and RNA expression in brain

Of the genes predicted to be alternatively spliced in ASD *versus* TD boys in this study, some have been implicated in previous genetic studies of ASD including Gelsolin (GSN), LRPPRC (leucine-rich PPR-motif containing), BIN1 (bridging integrator 1), MED12 (mediator complex subunit 12), NPAS2 (neuronal PAS domain protein 2), SYNE1 (spectrin repeat containing, nuclear envelope 1), and TBL1XR1 (transducin (beta)-like X-linked receptor 1) [73,89,95-97]. Thus, there is overlap of genes implicated in previous genetic ASD studies and the findings in this study of DAS in ASD.

Gelsolin has been implicated in previous genetic studies of ASD [95], has been reported to be differentially spliced in ASD compared to TD brain [5], and is predicted to be alternatively spliced in the ALL ASD *versus* TD analysis and the ASD_NTCV *versus* TD analysis in this study. Gelsolin protein binds the ‘plus’ ends of actin monomers and filaments to prevent monomer exchange, and modulates assembly and disassembly of actin filaments. Mutations of this gene cause familial amyloidosis Finnish type (FAF) [98]. Multiple transcript variants encoding several different isoforms have been found. Gelsolin levels increase in brain during development, and are higher in children with Down’s syndrome [99]. In brain gelsolin has many roles including modulating NMDA receptors, altering dendritic spines, being highly expressed by oligodendrocytes, and modulating amyloidosis [100-102].

The LPPRC gene has also been implicated in previous ASD genetic studies [73,95]. In the nucleus it binds HNRPA1-associated poly(A) mRNAs and is part of nmRNP complexes at late stages of mRNA maturation which are associated with nuclear mRNA export. In mitochondria the protein binds to poly(A) mRNA and stabilizes mitochondrially encoded cytochrome c oxidase (COX) subunits. It cooperates with PPARGC1A to regulate certain mitochondrially encoded genes and gluconeogenic genes and may regulate docking of PPARGC1A to transcription factors and regulates transcription of the multidrug-related genes MDR1 and MVP. The LPPRC proteinis reduced over two-fold in ASD compared to control brain [73]. The LPPRC protein directly binds Neurofibromin 1, mutations of which can be associated with ASD [103].

MED12 (see above) has also been implicated as a genetic susceptibility ASD gene [95]. A novel X-linked disorder with developmental delay and autistic features with duplication of Xq12-q13.3 involves the MED12 gene [104]. MED12 is linked to the sonic hedgehog pathway and mutations in the gene are associated with mental retardation and autism like features [86]. Med12-dependent recruitment of the Mediator complex with Sox10 promotes terminal differentiation of myelinating glia [105]. MED12 plays a key role in forming the hindbrain during development [84].

TBL1XR1 (transducin (beta)-like 1X-linked receptor 1 - trascription regulator, beta-catenin binding) has also been implicated in ASD. It localizes to the histone deacetylase complex and interacts with HDAC3 and RNA polymerase II, playing a key role in regulating transcription. This gene is one of six newly identified genes that have recurrent mutations that are associated with ASD [106]. This gene also regulates the beta catenin Wnt signaling pathway which has also been implicated in ASD [97,107].

Of the ASD genes found to demonstrate DAS in blood in this study, several of these have been reported to also be differentially alternatively spliced in ASD brain. These include: GSN; CLTB (clathrin, light chain); OS9 (osteosarcoma amplified 9, endoplasmic reticulum lectin); BIN1; CHPT1 (choline phosphotransferase 1); LSM14A (SCD6 homolog A); MINK1 (misshapen-like kinase 1); and SYNE1 [5]. Though the function of these genes may be different in blood and brain [108], and even though the exons involved are different as expected since alternative splicing is highly tissue-specific, DAS of the same genes in blood and brain represents an independent line of evidence for these genes being affected by DAS/DEU in different cohorts and tissues in ASD.

### Pathways previously implicated in ASD: subject-level pathways affected by DAS in ASD

The pathway analysis summarized in Figure 4 was based on the 21 pathways that were significantly different between ALL ASD and TD and which were derived from 477 exons predicted to participate in differential alternative splicing as well as have a significant difference of exon expression between ASD and TD. The unique feature of this analysis was that each pathway was scored as TD-like or non-TD like for each individual. The data show that no pathway is associated with all ASD children, and only five of 30 ASD children had alterations in all of the pathways, whereas the remainder had alterations in different subsets of pathways. The finding of alterations in the Natural Killer Cell and NGF pathways in the majority of ASD children confirms our and other previous studies [59-61]. Alterations in genes in monocyte-related pathways in most ASD children could relate to reported differential monocyte responses to TLR ligands in children with ASD [62], to microglia (brain macrophages) activation in ASD brain [22,109] and microglial genes being over-expressed in ASD brain [5].

Our pathway analyses per individual showed that 60% of the ASD children in this study had predicted DAS abnormalities of mTOR pathways (Figure 4). This is notable since mutations of single genes often associated with ASD clinical features also have aberrant mTOR signaling including Fragile X, tuberous sclerosis, PTEN, and neurofibromatosis [38,40]. Thus these data point to possible abnormalities of mTOR pathways in a subgroup of ‘idiopathic’ ASD which may have clinical relevance since mTOR inhibitors like rapamycin can modulate these pathways [110]. Though there are 21 pathways shared between all of the ASD subjects, and though some subjects share some common pathways, only five subjects share the same pathways [3]. Thus, a variety of different combinations of pathways appear to be associated with idiopathic ASD. This would be consistent with the many known genetic causes of ASD which are also associated with many different pathways. However, it also shows some convergence on similar biological processes at least for subgroups of ASD subjects as discussed above [111].

Our novel pathway analysis supports previously published data on alterations in pathways in ASD, such as oxidative stress [61], mTOR [38], Natural Killer cells [59,60], NGF, and monocyte pathways [112], as well as activation of microglia (macrophages in the periphery) [22,109]. However, this is the first study to suggest DAS/DEU occurs in these pathways and thus confirmation is needed in a future independent cohort. We performed this sub-analysis to address the heterogeneity in ASD at the level of each individual ASD subject. Though every gene/pathway may not be reproduced, we propose this as a plausible approach to investigate ASD heterogeneity and identify ASD subgroups at the transcriptome level.

### Differential alternative splicing in ASD with large TCV

There was one gene with DAS in ASD_LTCV when compared to TD: membralin (C19orf6). Membralin is a highly conserved transmembrane protein, which does not share significant sequence homology with other human genes, only membralins of other species. It is expressed in the central nervous system [113] and is associated with ovarian carcinoma [114]. Membralin interacts with UBC, TMEM173, and ARSE proteins (http://www.GeneCards.org; http://www.string-db.org). UBC, involved in protein degradation and ubiquitinization, was a convergence node of direct interactions with genes with DAS in All ASD *versus* TD and the LTCV *versus* NTCV ASD comparisons.

Though little is known about membralin function, some of its interacting partners belong to proteins implicated in ASD. A rare CNV in the AGMO (TMEM195) gene has been identified with autism in AGRE and NIMH cohorts [95,115]. In addition, a number of ubiquitin-related genes have also been implicated in ASD, and membralin interacts with the ubiquitin C (UBC) protein [95].

### Effect of total brain volume

One surprising result of the study was that though over 100 genes were predicted to demonstrate DAS for the ASD_NTCV *versus* TD comparison, only one gene was predicted to show DAS for the ASD_LTCV *versus* TD comparison (membralin). This could be interpreted to mean that DAS/DEU is associated more with the pathophysiology of ASD with normal brain volumes rather than ASD with large brain volumes. If this is the case, there is no ready explanation for this finding though it is consistent with a different pathophysiology of the ASD_LTCV and ASD_NTCV subgroups. There were 27 genes that demonstrated DAS in the ASD_NTCV *versus* ASD_LTCV comparison, which associated with many pathways including IL12 signaling in macrophages, Inositol Pyrophosphates Biosynthesis, synaptic long-term potentiation, PKA signaling, Dopamine-DARPP32 Feedback in cAMP Signaling, and others. Given that only one gene differed in the ASD_LTCV to TD comparison, these differences are likely driven by the ASD_NTCV group. The data would suggest that future genetic, imaging, biomarker, and behavioral studies should consider head size for defining clinical ASD subgroups.

### Limitations

Cause and effect cannot be determined from this study. Since blood samples were used, it is not known to what extent the observed changes reflect changes in the brain. It is likely that many of these changes reflect differences in the peripheral immune system of ASD and are associated with the immune and autoimmune dysregulation observed in some ASD subjects [112].

The sample size is small and though multiple comparison adjustments were made, the problem of false positives can only be addressed by replication in future studies. CNVs, SNPs, and other processes could affect the exon usage measured in this study which would not represent differences of alternative splicing. Therefore, alternative splicing predicted using exon arrays in this study will require confirmation using other approaches that directly measure expression of alternatively spliced variants of single genes. The current study provides one source for determining which genes might be evaluated in future studies.

## Conclusions

The data in this study suggests that DAS occurs in blood of 2-4-year-old boys with ASD compared to TD controls. Different ASD subgroups based on TCV exhibited specific DAS. These findings are preliminary and need to be replicated in independent cohorts.

## Abbreviations

ASD: Autism spectrum disorders; DAS: Differential alternative splicing; DEU: Differential exon usage; LTCV: Large total cerebral volume; NTCV: Normal total cerebral volume; TD: Typically developing.

## Competing interests

The authors declare that they have no competing interests.

## Authors’ contributions

BS and FRS devised the study. CWN, MDS, SR, and DGA selected participants, oversaw diagnostic evaluations, and directed MRI studies and analyses. BS and YT performed the statistical analyses. BS and FRS wrote the manuscript and all authors participated in reviewing and editing the manuscript.

## Supplementary Material

Additional file 1: Figure S1 Principle components analysis (PCA) on a whole-genome level of all samples included in our analyses. Color-coding based on Group: TD - blue, ASD_NTCV - orange, ASD_LTCV - gray. **Figure S2.** IPA top-scoring networks of the 53 genes with DAS/DEU (ALL ASD *vs.* TD): Free Radical Scavenging , Cell Death and Survival, Small Molecule Biochemistry. Red - predicted exon with DAS/DEU is more often retained in ASD than in TD; green - predicted exon with DAS/DEU is more often excluded in ASD than in TD. Solid lines represent direct interactions. Dashed lines - indirect interactions. Note: PI3K node, which displays DAS. Colors represent genes predicted to display DAS/DEU between ALL ASD and TD. **Figure S3.** IPA second top-scoring networks of the 27 genes with DAS/DEU in ASD_LTCV *vs.* ASD_NTCV: Cellular Assembly and Organization, Cellular Function and Maintenance, RNA Post-Transcriptional Modification. Note: UBC convergence hub of direct interactions. Colors represent genes predicted to display DAS/DEU between ASD_LTCV and ASD_NTCV and TD. Red - predicted exon with DAS/DEU is more often retained in ASD_LTCV than in ASD_NTCV; green - predicted exon with DAS/DEU is more often excluded in ASD_LTCV than in ASD_NTCV. Solid lines represent direct interactions. Dashed lines - indirect interactions. Click here for file

Additional file 2: Table S1 Genes (*n*=53) with predicted DAS/DEU in ALL ASD *vs.* TD groups (FDR *P* <0.05). **Table S2.** Genes (*n*=169) with predicted DAS/DEU in ASD_NTCV *vs.* TD groups (FDR *P* <0.05). **Table S3.** Genes (*n*=27) with predicted DAS/DEU in ASD_LTCV *vs.* ASD_NTCV groups (FDR *P* <0.05). **Table S4.** Genes (*n*=477) with predicted DAS/DEU in sub-analysis of ALL ASD *vs.* TD Groups for individual subject-level pathway analysis. **Table S5.** Functional analysis using IPA of the 477 DAS genes in the sub-analysis of ALL ASD compared to TD (see Table S4) revealed that 21 canonical pathways were over-represented (*P* <0.05). These 21 pathways and the genes with DAS in each pathway are listed below. These 21 pathways were used for the analysis shown in Figure 4. Click here for file
