# Supplementary material for: Evidence for differential alternative splicing in blood of young boys with autism spectrum disorders
Source: Mol Autism. 2013 Sep 4;4:30. doi: 10.1186/2040-2392-4-30 (PMC3846739; doi:10.1186/2040-2392-4-30)
Supplement: Additional file 1: Figure S1 — Principle components analysis (PCA) on a whole-genome level of all samples included in our analyses. Color-coding based on Group: TD - blue, ASD_NTCV - orange, ASD_LTCV - gray. Figure S2. IPA top-scoring networks of the 53 genes with DAS/DEU (ALL ASD vs. TD): Free Radical Scavenging , Cell Death and Survival, Small Molecule Biochemistry. Red - predicted exon with DAS/DEU is more often retained in ASD than in TD; green - predicted exon with DAS/DEU is more often excluded in ASD than in TD. Solid lines represent direct interactions. Dashed lines - indirect interactions. Note: PI3K node, which displays DAS. Colors represent genes predicted to display DAS/DEU between ALL ASD and TD. Figure S3. IPA second top-scoring networks of the 27 genes with DAS/DEU in ASD_LTCV vs. ASD_NTCV: Cellular Assembly and Organization, Cellular Function and Maintenance, RNA Post-Transcriptional Modification. Note: UBC convergence hub of direct interactions. Colors represent genes predicted to display DAS/DEU between ASD_LTCV and ASD_NTCV and TD. Red - predicted exon with DAS/DEU is more often retained in ASD_LTCV than in ASD_NTCV; green - predicted exon with DAS/DEU is more often excluded in ASD_LTCV than in ASD_NTCV. Solid lines represent direct interactions. Dashed lines - indirect interactions. [file 2040-2392-4-30-S1.pdf]

Supplementary Figures

Differential Splicing in Autism

Stamova et al.

Figure S1.

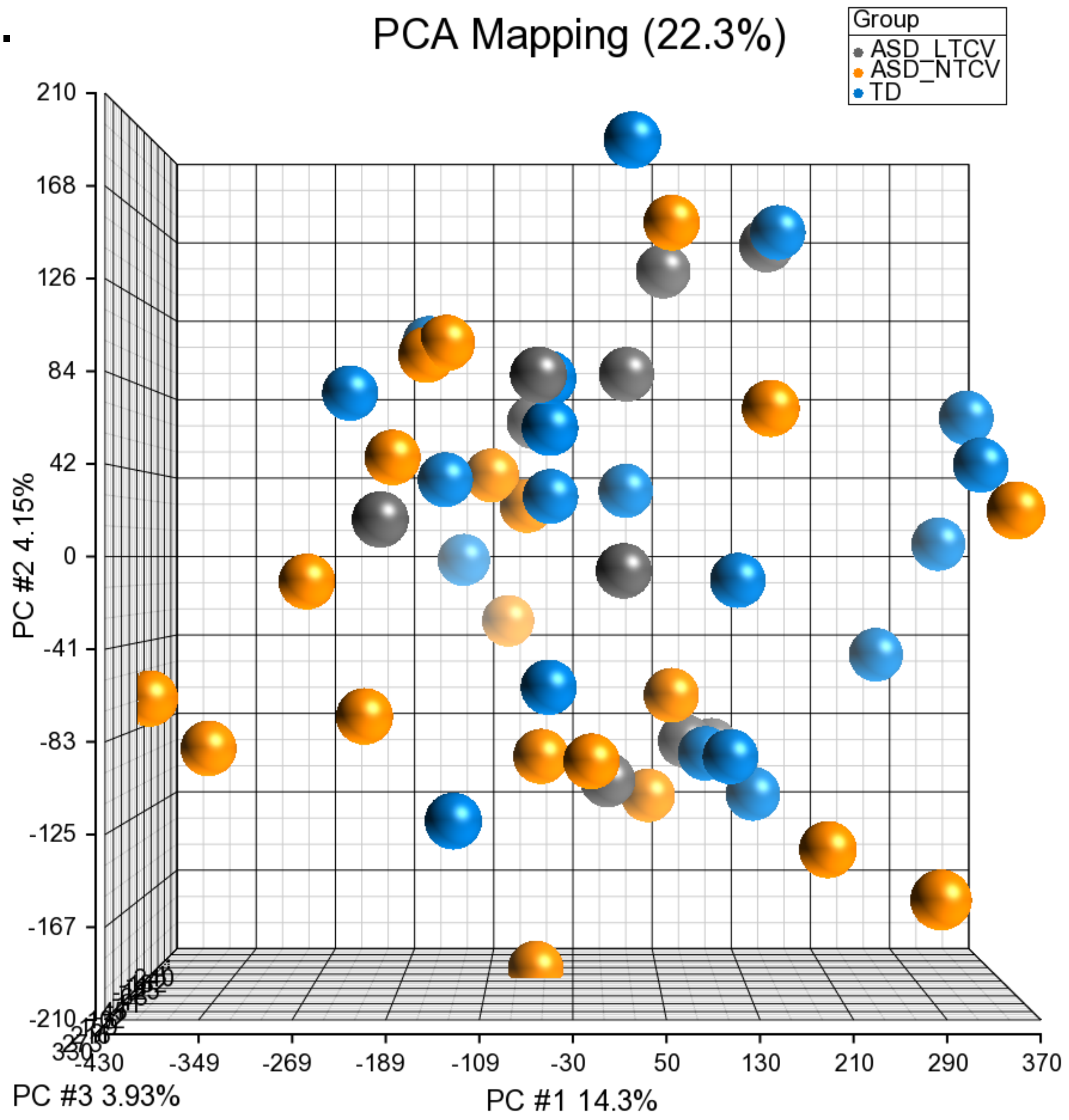
PCA Mapping (22.3%)
Group
ASD_LTCV
ASD_NTCV
TD
210
168
126
84
42
0
-41
-83
-125
-167
-210
PC #2 4.15%
-430
-349
-269
-189
-109
-30
50
130
210
290
370
PC #1 14.3%
PC #3 3.93%

Figure S2.

Path Designer Network 1

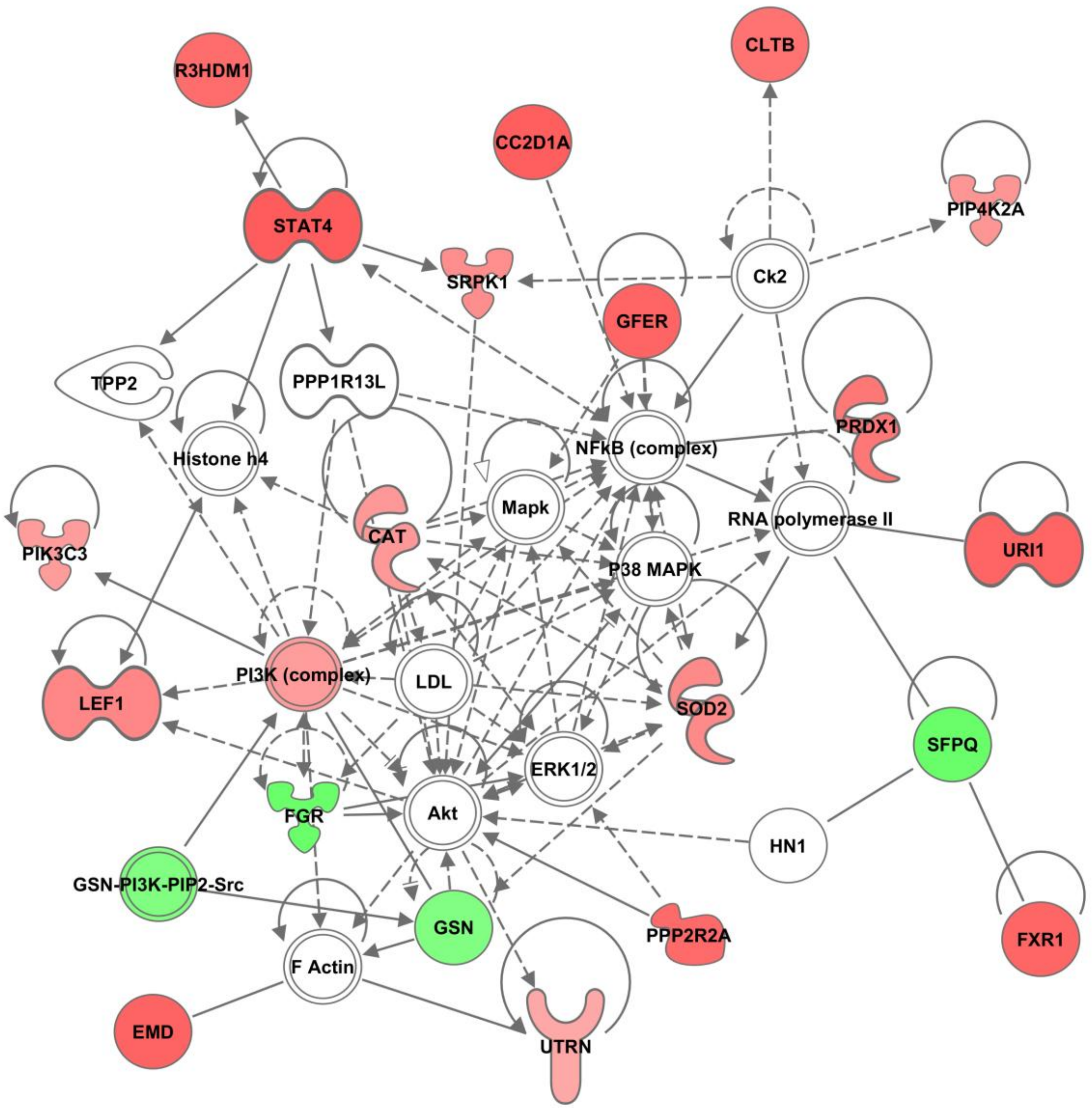

© 2000-2013 Ingenuity Systems, Inc. All rights reserved.

Figure S3.

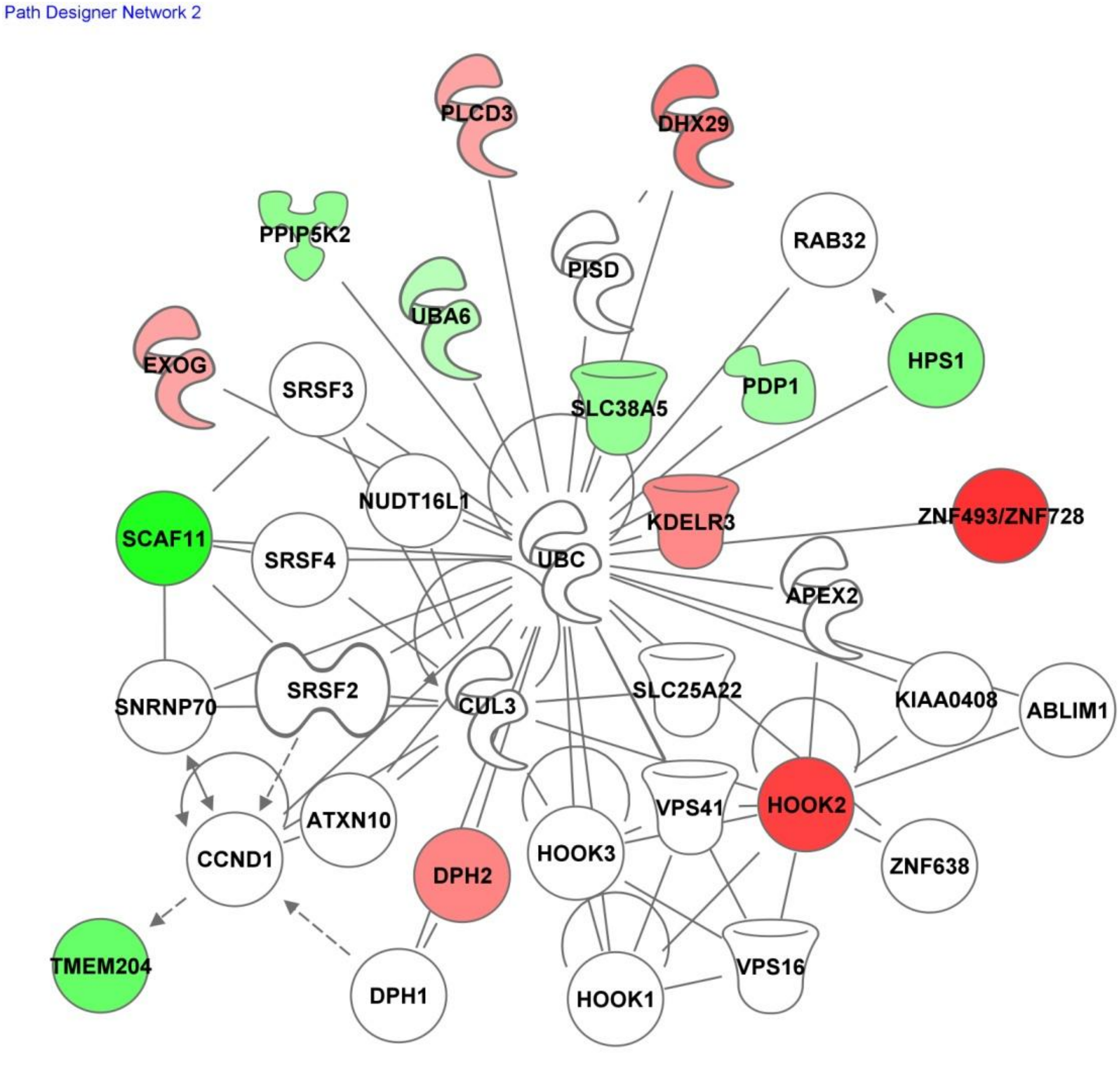

© 2000-2013 Ingenuity Systems, Inc. All rights reserved.
